# goFOOD^TM^: An Artificial Intelligence System for Dietary Assessment

**DOI:** 10.3390/s20154283

**Published:** 2020-07-31

**Authors:** Ya Lu, Thomai Stathopoulou, Maria F. Vasiloglou, Lillian F. Pinault, Colleen Kiley, Elias K. Spanakis, Stavroula Mougiakakou

**Affiliations:** 1 ARTORG Center for Biomedical Engineering Research, University of Bern, 3008 Bern, Switzerland; ya.lu@artorg.unibe.ch (Y.L.); thomai.stathopoulou@artorg.unibe.ch (T.S.); maria.vasiloglou@artorg.unibe.ch (M.F.V.); 2 Division of Endocrinology, Baltimore Veterans Administration Medical Center, Baltimore, MD 21201, USA; lillian.pinault@va.gov (L.F.P.); ispanakis@som.umaryland.edu (E.K.S.); 3 Luminis Health, Anne Arundel Medical Center, Anne Arundel Medical Group Diabetes and Endocrine Specialists, Annapolis, MD 21401, USA; ckiley@aahs.org; 4 Division of Endocrinology, Diabetes, and Nutrition, University of Maryland School of Medicine, Baltimore, MD 21201, USA; 5 Bern University Hospital “Inselpital”, 3010 Bern, Switzerland

**Keywords:** carbohydrate, protein, fat, calorie, nutrient estimation, computer vision, smartphone

## Abstract

Accurate estimation of nutritional information may lead to healthier diets and better clinical outcomes. We propose a dietary assessment system based on artificial intelligence (AI), named goFOOD^TM^. The system can estimate the calorie and macronutrient content of a meal, on the sole basis of food images captured by a smartphone. goFOOD^TM^ requires an input of two meal images or a short video. For conventional single-camera smartphones, the images must be captured from two different viewing angles; smartphones equipped with two rear cameras require only a single press of the shutter button. The deep neural networks are used to process the two images and implements food detection, segmentation and recognition, while a 3D reconstruction algorithm estimates the food’s volume. Each meal’s calorie and macronutrient content is calculated from the food category, volume and the nutrient database. goFOOD^TM^ supports 319 fine-grained food categories, and has been validated on two multimedia databases that contain non-standardized and fast food meals. The experimental results demonstrate that goFOOD^TM^ performed better than experienced dietitians on the non-standardized meal database, and was comparable to them on the fast food database. goFOOD^TM^ provides a simple and efficient solution to the end-user for dietary assessment.

## 1. Introduction

Diet-related diseases—such as cardiovascular diseases and diabetes—are the leading causes of death globally. Macro-vascular diabetes complications such as atherosclerotic cardiovascular disease are also the most common cause of morbidity and mortality for individuals with diabetes [1], while the estimated cost for care of diagnosed diabetes accounts for 25% of health related expenses in the USA in 2017 [2]. For individuals living with cardiovascular diseases, a balanced diet which is low in saturated and trans-unsaturated fat and high in fruits and vegetables, can reduce the risk of ischemic heart disease and stroke. People with diabetes need to monitor their diet, specifically their carbohydrate (CHO) intake, as it is a key factor that can affect blood glucose levels. Clinical studies on insulin dependent children and adolescents have shown that an error of ±20 grams in CHO estimation has significant effects in controlling postprandial glycaemia [3]. These individuals have to receive training on CHO counting, which relies on empirical rules. This results in errors in their estimation, ranging from 10 to 15.4 grams [4,5,6]. Moreover, other diseases, such as obesity, certain types of cancer, osteoporosis and dental diseases, have been associated by the World Health Organization (WHO) with diet and nutrition [7]. All these diseases require monitoring and assessment of the individual’s diet to different extents and for different reasons.

With the development of computer vision algorithms and smartphone technologies, it has become feasible to estimate the nutrient content of the food by analysing meal images captured from the smartphone camera [8,9,10]. In an ideal scenario, the users need to capture one or more meal images using the smartphone camera; the food type and the associated nutrient contents will be automatically calculated by the dedicatedly designed dietary assessment system. Three stages are normally involved in such systems: (1) food item segmentation; (2) food item recognition and (3) volume estimation. Thus, the nutrient content can be retrieved using the food nutrient database in a straightforward manner.

Following this concept, many algorithms have been proposed for dietary assessment [11,12,13,14,15], but most of these only focus on the first two steps [11,12,13], using the associated image segmentation and recognition algorithms. Even though these algorithms achieve good accuracy on the publicly available databases [16,17], we observed that it is still difficult for them to provide satisfactory performance on real life images, especially in the case of blurred images or poor lighting conditions. Thus, for a practical system a human-interaction module must be implemented to enable the end users to manually correct the automatically generated food segmentation and recognition results.

Another major challenge of the dietary assessment system lies within the estimation of food volume. Conventionally, the multi-view geometry-based approach [18,19] is applied to build the food 3D model for volume estimation. The approach requires multiple carefully captured input images and a rather large reference pattern (e.g., a reference pattern with a size similar to the plate’s), making it difficult to use in practice. Our previous work, GoCARB [15,20,21], optimises such an approach using the dedicatedly designed camera pose estimation and stereo-matching strategies, and it requires only two free-angle food images and a credit card-sized reference card as input. The GoCARB system is an initial attempt to achieve practical estimation of the food volume in real scenarios and has been validated both technically [22] and in a framework of pre-clinical and clinical trials [15,23]. Following the development and great progress of the CNNs, a number of recent studies have tried to address the estimation of food volume using single view colour images [14,24,25,26]. Ref. [14,26] uses the CNNs predicting the depth image from single-view colour image, while the predicted depth map is used for the food volume calculation. Ref. [24,25] treats the food volume as a latent variable and predicts the food nutrient content directly from the colour image using the CNNs. Although such approaches achieve ultimate convenience for the end-users, the methods themselves are ill posed and are therefore difficult to generalize in real life. Moreover, such approaches require large amount of training data with depth image or food nutrients as ground truth, which is expensive and difficult to acquire. To this end, at the current stage, we believe that the two-view geometry-based approach (i.e., the solution of GoCARB) is adequately practical for accurate dietary assessment.

The progress in academic work on AI-based dietary assessment has been accompanied by several attempts to commercialise the technology in smartphone applications (e.g., FatSceret [27], CALORIE MAMA [28] and bitesnap [29]). These applications take advantage of the availability of smartphones and AI algorithms and aim to provide convenient tools for general users to regularly record their meals. However, the majority of the applications require the user to manually estimate the food portion size [27,28,29] or to use standardised portion units [27,28,29]. Our recent research indicates that even trained individuals cannot estimate the food portion accurately [23]. Furthermore, to the best of our knowledge, the commercially available applications are not supported by publications that present information on (i) the algorithmic methods used, (ii) validation-even in controlled conditions or using a benchmarked dataset and (iii) the system architecture (e.g., smartphone based, server-based). Therefore, comparative assessment is not straightforward.

In this paper, we propose a smartphone-based dietary assessment system, named goFOOD^TM^. goFOOD^TM^ follows a similar pipeline as our previous GoCARB system, which estimates the nutrient contents of the food by using two input images. However, the following modifications have been made: (1) goFOOD^TM^ takes advantage of the specially designed advanced deep learning method, ensuring that the system supports more food types and with better accuracy. (2) In addition to CHO, goFOOD^TM^ also estimates Protein (PRO), Fat and calorie content of the food. (3) The gravity data from the Inertial Measurement Unit (IMU) of the smartphone is used to further optimize camera pose and table plane estimation in the food volume estimation module. (4) goFOOD^TM^ supports both two-view images and the stereo image pair for a smartphone equipped with two rear cameras as input. (5) A lighter version has also been developed, namely goFOOD^TM^Lite, which does not implement macronutrient and calorie estimation, but is used to simply record and store the user’s meal.

The performance of goFOOD^TM^ is evaluated on two databases: (1) MADiMa database [30] and (2) a new database named “Fast food” database, which contains images of food from the “McDonald’s” fast food chain. It should be noted that the MADiMa database only contains food images captured from different angles of view using single camera smartphones, while the “Fast food” database includes both two-view images (single camera smartphones) and stereo image pairs captured by smartphones with two rear cameras. To demonstrate the performance of the proposed system more convincingly, we also compare the output of goFOOD^TM^ to the estimations of two registered dietitians from the USA on both databases. The study results show that the proposed system performs better than the dietitians’ estimations on the MADiMA database, while similar performance is shown on the “Fast food” database. In addition, to illustrate the advantages of the proposed system with respect to the typical commercial dietary assessment systems, we summarize a high-level comparison between the proposed goFOOD^TM^ and some popular commercial applications in Appendix A.

Our contributions can be summarised as follows:

1. We propose a practical, accurate, smartphone-based dietary assessment system that predicts the macronutrient (CHO, PRO, Fat) and calorie content of a meal using two images. The experimental results demonstrate the superior performance of the proposed system with respect to the state-of-the-arts on two food databases.
2. A new database (“Fast food” database) is introduced. The database contains both food images captured from different views and stereo image pairs. Moreover, the accurate nutrient ground truth and the estimations of the dietitians are also provided. We plan on making this database publicly available to contribute to the dietary assessment research society.
3. We have conducted a study that compares our system’s estimation to the estimations of experienced dietitians, demonstrating the promising advantage of an AI-based system for dietary assessment.

## 2. System Outline

**Figure 1:**
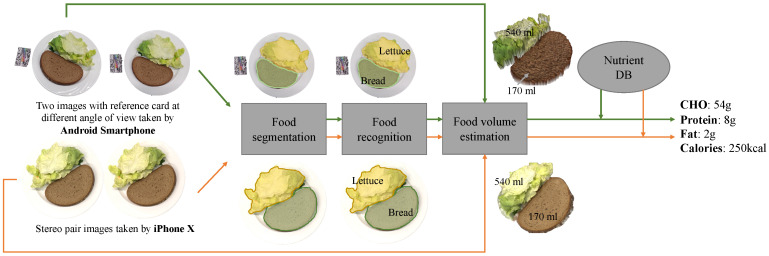
Overview of goFOOD^TM^.

The system requires the input of two meal images. The input images can be acquired either by conventionally capturing photos, or by recording a video. For conventional single-camera smartphones, the images must be captured from two different viewing angles; however, smartphones equipped with two rear cameras require only one press of the shutter button. The deep neural networks are applied to process the two images, and this performs food segmentation and recognition, while a 3D reconstruction-based algorithm estimates food volume. Each meal’s calorie and macronutrient content are calculated on the basis of each food category, volume and the a food composition database (Figure 1).

### 2.1. Food Image Acquisition

**Figure 2:**
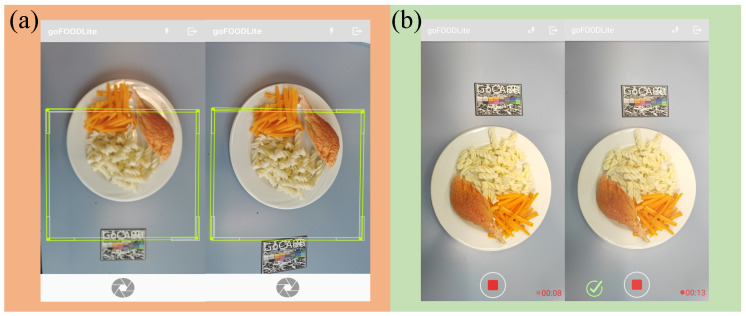
The application: (**a**) goFOOD^TM^Lite—two images capturing (**b**) goFOOD^TM^Lite—Video recording.

For food image acquisition, two methods using the smartphone’s camera have been developed and are currently in use. The first is plain photo capture as mentioned above, and requires the user to capture 2 images from specific angles (see Figure 2). When the smartphone is equipped with at least two cameras, the relative position of the cameras is known and this allows the user to press the shutter button only once: two photos are effectively captured simultaneously. For a single camera phone, the user must capture the two photos separately, while the app indicates the correct angle from which each photo must be captured, namely 90${}^{{}^{\circ}}$ and 75${}^{{}^{\circ}}$ from the table’s plane. These angles are selected on the basis of previous research [20], which indicates that the pair is optimal for volume reconstruction. The second method is video recording. The user is required to record a short video while moving their smartphone, so that as many different angles as possible are captured within the video clip.The application, while the video is recorded, automatically captures the most appropriate stereo-pair image, at angles 90${}^{{}^{\circ}}$ and 75${}^{{}^{\circ}}$ from the table’s plane. Once the appropriate data have been collected, the user is notified and the recording can stop.

For the current system, many prerequisites regarding data capturing have been eliminated, yet some remain necessary. In the case of a single camera smartphone, for other than the specific angles from which the images need to be captured, the user needs to place a credit card-sized reference object upon the surface or table. The food is not required to be placed on a plate, since the system can identify food on its own. It is however required that the meal (either plated or not) is placed upon a flat surface (the reference object needs to be placed on the same surface). This requirement is needed for the proper volume estimation. Finally, there is no prerequisite with respect to both texture and colour of the surface/background. However, a surface which exhibits high contrast to the food or plate, both texture- and color-wise guarantees better and easier segmentation.

The procedure for image acquisition supports goFOOD^TM^, but also exists as a standalone application, called goFOOD^TM^Lite. With the standalone version, it is possible to focus only on large-scale data gathering to create a larger database for the further development and evaluation of goFOOD^TM^.

### 2.2. Food Segmentation

**Figure 3:**
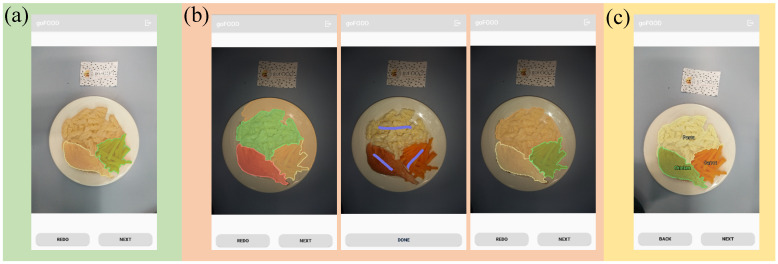
The application: (**a**) goFOOD^TM^—Successful automatic segmentation; (**b**) goFOOD^TM^— Failed automatic segmentation due to bad lighting [**left**]—Manual user input [**middle**]— Successful semi-automatic segmentation [**right**]; (**c**) goFOOD^TM^—Automatic Recognition.

goFOOD^TM^ supports both the automatic and semi-automatic modes for food segmentation. Automatic segmentation is performed immediately after the system receives the meal-related data (Figure 3a) using the embedded image segmentation algorithm. The user needs to check whether the segmentation result from the automatic mode is correct. If the segmentation result is unsatisfactory, they are advised to use the semi-automatic mode. The user should indicate each food item on the image by touching the corresponding area on the smartphone screen, as shown in Figure 3b. Then a new segmentation map is generated by the system, in accordance with both the user’s input and the embedded segmentation algorithm.

The algorithm used for the automatic food segmentation is based on the instance segmentation approach. More precisely, we are using the Mask-RCNN framework [31] in the proposed system. Even though the Mask-RCNN is able to address both the food segmentation and recognition simultaneously, we only use it to deal with the food segmentation task. We do this because collecting the training database with instance segmentation ground truth for a large amount of food categories is costly and unpractical. Thus, at this point we do not distinguish the different food types, which allows us to take advantage of the publicly available databases for the food segmentation network training [11,12]. For the semi-automatic food segmentation, the traditional region growing and merging [20] algorithm is applied. The interaction regions from the end-users (e.g., the purple lines in Figure 3b) are used as the seed points of the algorithm [20].

### 2.3. Food Recognition

**Figure 4:**
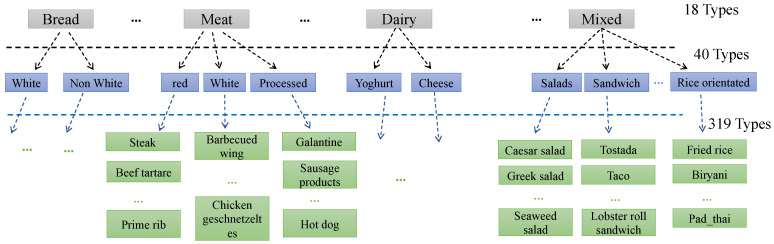
The food categories are organized in a three-level hierarchy. The green labels indicate fine-grained food categories supported by the system, while the gray and blue labels are the concluded first and second level hyper food categories, respectively.

goFOOD^TM^ supports 319 preinstalled, fine-grained food categories. Even though goFOOD^TM^ recognizes the most frequent food categories, in real-life it is still impossible for the system to recognize all the food categories existing in the world, and this is also the limit for all existing applications so far. To alleviate this issue, a hierarchical architecture of three levels has been designed, with each sub-category containing a larger number of more specific categories (Figure 4). In this way, an additional two-level hyper food category is assigned to each fine-grained food category and the resulting number of hyper food categories is relatively limited in the real world. If an unknown fine-grained food type is detected, goFOOD^TM^ is able to output the hyper food category correctly for the approximate estimation of food nutrient. Moreover, this hierarchical architecture of food category representation can also improve the system’s recognition accuracy comparing with the normally used one-level food label representing method (which is demonstrated in Section 3).

The convolutional neural networks [32] are designed and implemented for hierarchical food recognition. Practically, any kind of prevalent CNN for image classification can be used as the backbone of our food recognition module. Here we choose the Inception V3 [32] due to its high accuracy in food classification task [33]. We replace the last fully connected layer of the Inception-V3 with three parallel food recognition layers that correspond to our three levels of food categories. The input of the algorithm is the food image that is trimmed by the segmentation map from the previous step, while the outputs are the two-level hyper- and fine-grained categories. A weighted inference strategy is applied to the three tags and the one with the highest confidence for the nutrient content calculation is the final output. The training dataset consists of images collected from the Internet, from publicly available databases [16,34] and the internally captured meal images. As with food segmentation, the semi-automatic mode is also supported in the food recognition module (Figure 3c), which permits the user to manually modify the food category when the results from the automatic mode are not satisfactory.

### 2.4. Food Volume Estimation

As mentioned before, the input data are either from two views with a traditional single-camera smartphone (from two single images or a video capture) or a stereo-image pair using a smartphone with two rear cameras. Different strategies are applied for these two scenarios to estimate the food volume. For the case of different-view images, a reference object of known size must be placed next to the food when taking photos (this is not required in the case of two rear cameras). In order to reconstruct the food 3D model, we use a similar method as in our previous work [20]. However, we further improve this method by using the gravity data from the smartphone’s IMU. The orientation of the plate’s bottom is retrieved directly from the gravity data by assuming the table plane is horizontal in the world coordinates. Moreover, the camera rotation along the smartphone edges is calculated using the rotation of the gravity vector instead, which significantly reduces the computational complexity for the camera pose estimation and gives a more stable performance.

### 2.5. Nutrient Estimation

The calories and macronutrient content of each food category (per 100 ml) is retrieved from the “Nutritionix Database” [35], an online nutritional database, which includes the nutrient content of almost 885 K food types. With this database and the estimated food volume, a straightforward calculation gives the nutrient content of the meal. As can be seen in Appendix C, the definition of the categories is based not only on the visual characteristics of the food, but also its nutritional content. Salient differences are therefore distinguished between the recognised foods, such as different pasta dishes using the same type of pasta but different sauce, which could substantially affect the final nutrient content. Moreover the categories cover a large range of dishes and cuisines, constituting the nutrient estimation appropriate for usage in different locations.

### 2.6. Pipeline Setup

In our attempt to minimize the hardware requirements of the smartphone, no data are kept and no calculations are implemented locally on the smartphone. The captured images and/or recorded videos are immediately transmitted and stored in our servers, where our application programming interface (API) is implemented and in function. The smartphone interacts with the server through a series of HTTP requests and responses and receives all intermediate results, as well as the final estimation. This data is not stored on the phone.

## 3. Experimental Analysis

The objective of goFOOD^TM^ is to reduce the effort of the average user when it comes to dietary assessment, while achieving performance comparable to healthcare professionals. In this section, we evaluate the performance of goFOOD^TM^ by comparing it to both the ground truth and dietitians’ estimations.

### 3.1. Evaluation Databases

Two food image databases were used during our experiments:

**MADiMa database [30]:**

The database contains 80 central-European style meals. Each meal contains 2–4 food items that were carefully weighed and annotated with semantic segmentation map and volume. The database includes 234 food items and 64 fine-grained food types. It should be noted that the original database annotated the 234 food items with 22 food types. We re-annotated the data in accordance to the food categories described earlier, in order to align the database with goFOOD^TM^. Each meal in the database contains the images captured from different viewing angles (between 30–90${}^{{}^{\circ}}$ with the table plane) and distance (40–60 cm to the table) using multiple sensors (smartphone camera and Intel Realsense depth camera). The pixel-level semantic map of the meal images are manually annotated and the ground truth calorie and macronutrient content for each food item are calculated from the recorded food weight and food composition databases (USDA [36] and/or Swiss [37]. More details regarding the database can be found in [30]).

The MADiMa database was used to evaluate the performance of the full pipeline of the dietary assessment, i.e., food segmentation, recognition, and estimations of volume and nutrient content. To fairly compare our goFOOD^TM^ system with the state of the art ones [30,38], we only employ the colour images captured by smartphone, at 40 cm, 90${}^{{}^{\circ}}$ and 60${}^{{}^{\circ}}$ for evaluation (according to [20], the 90${}^{{}^{\circ}}$ and 75${}^{{}^{\circ}}$ (or 70${}^{{}^{\circ}}$) are the most accurate combination for food volume estimation. However, the MADiMa database does not contain such combination, we therefore chose the 90${}^{{}^{\circ}}$ and 60${}^{{}^{\circ}}$ as the other works [30,38]).

**Fast food database:**

The MADiMa database only contains meal images captured by monocular cameras, while goFOOD^TM^ supports both two view images and stereo-image pairs as input. To thoroughly evaluate the performance of goFOOD^TM^, a new database that contains both two view images and stereo image pairs of each meal was constructed, using food from the international fast food chain“McDonald’s”.

**Figure 5:**
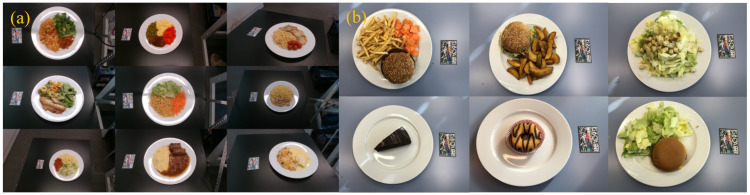
Some example meal images in (**a**) MADiMa and (**b**) Fast food Databases.

This database contains 20 meals and includes 14 different food types. Each meal contains 1–3 food items. The two-view images and stereo image pairs for each meal were captured using an Android smartphone (with goFOOD^TM^Lite) and an iPhone *X*, respectively. The ground truth calories and macronutrient content were retrieved from the official website of “McDonald’s”. Figure 5 exemplifies some meal images contained in both MADiMa and Fast food databases.

### 3.2. Dietitians’ Estimation

Two dietitians from the USA, with over 5 years of experience in macronutrient counting, participated in the study. They were asked to perform visual estimations for macronutrients (in grams) and calories (in kcal) for each meal and report them on a dedicated excel file (both for the MADiMa and the fast food databases). A detailed description of the estimation method followed by the dietitians is described in Appendix B.

## 4. Results

### 4.1. Food Image Processing

The evaluation of food image processing included food segmentation, recognition and volume estimation. Although both automatic and semi-automatic modules are implemented in goFOOD^TM^ for the first two aspects, we only evaluated the system’s performance under automatic mode. The performance of the semi-automatic mode heavily depends on the user, which is not objective enough for the evaluation. For the food volume estimation, the ground truth segmentation map was applied for the food 3D model generation.

**Figure 6:**
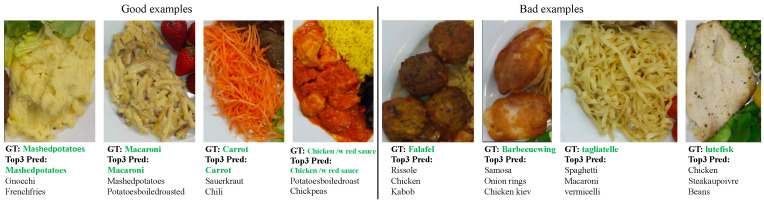
Examples of correctly and incorrectly recognized food images.

We applied the same metric as described before [39] (i.e., F-score) for the evaluation of food segmentation. Higher F-scores indicate better segmentation performance. Table 1 compares the results of the food segmentation. goFOOD^TM^ achieved 94.4% of the $F_{sum}$ and 83.9% of the $F_{min}$ on the MADiMa database, which are higher than the other works in the literature [30,39], and indicates that the good performance of food segmentation module of goFOOD^TM^. Table 2 reveals the top-1 and top-3 accuracy in all the three-level food classification tasks (2 hyper- and 1 fine-grained). To demonstrate the advantage of the joint three-level prediction architecture of goFOOD^TM^, we compare our result with the original Inception-V3 [32] networks, by implementing three individual Inception-V3 networks for each level of food category respectively. The comparison was conducted under the same experimental conditions: The training data was augmented using the same way as [40]; The stochastic gradient descent (SGD) optimizer is applied with the initial learning rate 1 × 10${}^{-2}$; The batch size is set as 32 and the number of epochs is 20. From the results in Table 2, the joint learning architecture (i.e., goFOOD^TM^) outperforms the original network regarding the top-1 accuracy for all the food category levels, demonstrating the good performance of the proposed multi-level food classification strategy. It should be noted that the evaluation process was conducted with one image crop. Although the multi-image crops and embedded backbone models improve the recognition accuracy, such strategies are not practical for real-life applications due to high computational cost. Figure 6 shows some typical examples of correctly and incorrectly recognized images. We can see that the errors are mainly due to the similar visual appearance of the inter food categories. For example, the “falafel” (a type of fried ball made from ground chickpeas), looks very similar to the “Rissole”, which is a kind of fried meat ball. The “lutefisk” in Figure 6 was easily misidentified as a piece of chicken breast.

The computation time of the whole food image processing module is ∼2.7 s on a server equipped with GTX1080Ti and i7−4770KCPU@3.5GHz. We evaluated the food volume estimation as in [20], which gave a 19% Mean Absolute Relative Error (MARE) over the 234 food items in the MADiMa database. In comparison to our previous GoCARB system (which has 22.6% MARE), the improvement in performance is mainly due to the introduction of the gravity data from the smartphone.

### 4.2. Nutrient Estimation

Table 3 and Table 4 compare the results of the goFOOD^TM^ system and the dietitians’ estimations on both the MADiMa and the Fast Food databases. The metric we used for evaluation was the median of the absolute error and its 25th and 75th percentiles.

As indicated by the results, the proposed goFOOD^TM^ system achieved satisfactory results on both databases. It should be noted that goFOOD^TM^ performed much better than the dietitians’ estimations on the MADiMa database, and was somewhat inferior to the dietitians’ estimations on the Fast Food database. To further support the above findings, the two-sample t-test is conducted on both databases (statistical significance was set to 0.05). The null hypothesis was set as: “the dietitian’s estimation is closer to the ground truth than goFOOD^TM^’s estimation”. Examining the results, the *p*-values of CHO, PRO, Fat and calories on the MADiMa database are all lower than 0.05, indicating that the null hypothesis can be rejected and the alternative, i.e, *goFOOD^TM^ has closer performance to the ground truth than the dietitians on the MADiMa database*, should be accepted. However, the *p*-values of CHO, PRO, Fat and calories on the Fast food database are 0.062, 0.224, 0.052, 0.054, respectively. Thus, on the Fast food database we accept the null hypothesis, that the dietitians’ estimation are closer to the ground truth than goFOOD^TM^.

We believe that this occurred due to the experiences of the dietitians. We theorize that they, like most people, are well familiarized with standardized meals (standard portions, plate sizes, nutrient contents etc.), that are common in fast food chains, such as McDonald’s. On the other hand, when it comes to everyday meals from individuals, the diversity is much greater, regarding all parameters that can affect the dietary estimation of a meal from just an image (portion sizes, plate sizes, food combinations etc.). Moreover the current study involved populations, and thus meals, from Central Europe, which can pose an extra obstacle for the dietitians originating from the USA, due to differences in culinary culture. These factors pose greater challenges for a dietary estimation, which is indicated by the experimental results.

In Table 4, the performance of the two different system inputs (i.e., two different view images and stereo image pair) are also compared. As shown by the results, the input of two view images achieved better results, which is due to the large baseline distance between the two view images (∼20 cm), while that of the stereo-image pair is very small (∼14 mm for the iPhone X). The small baseline distance then has a negative effect on the 3D model reconstruction [41].

The *Pearson* correlations between goFOOD^TM^, the dietitians’ estimations and the ground truth are illustrated in Table 5. As indicated by these numbers, the estimations of goFOOD^TM^ were correlated with both the ground truth values and the dietitians’ estimations. The highest correlation values (>0.6) are found between the PRO, fat and calorie estimations and the ground truth from the MADiMa database and between the CHO, PRO and calorie estimations and the ground truth from the Fast Food database.

**Figure 7:**
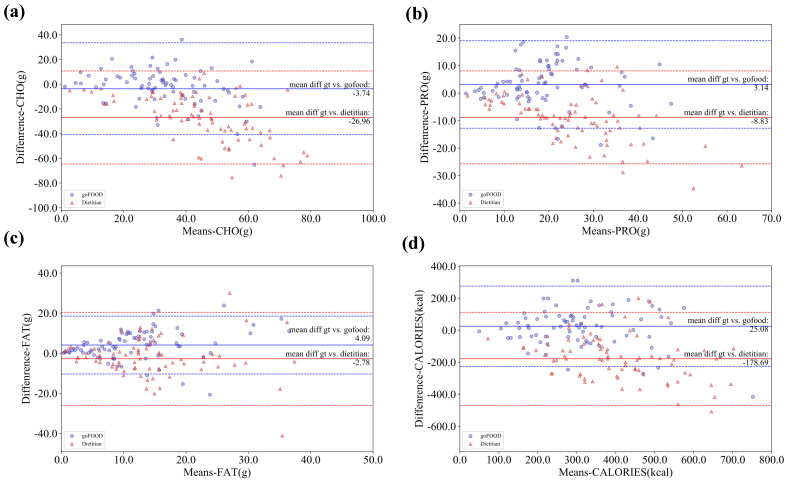
Bland–Altman plots of goFOOD^TM^’s and dietitians’ estimations on the MADiMa database in terms of (**a**) CHO, (**b**) PRO, (**c**) FAT and (**d**) Calories. The dashed lines indicate the 95% confidence interval of goFOOD^TM^ (blue) and the dietitians’ estimations (red).

**Figure 8:**
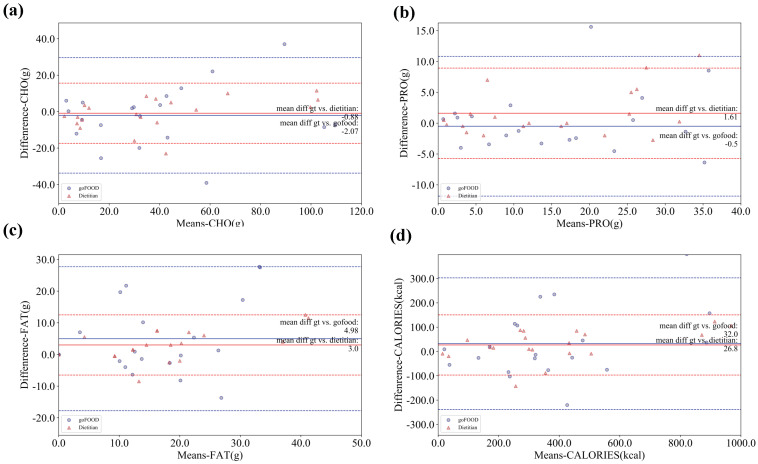
Bland–Altman plots of goFOOD^TM^’s and dietitians’ estimations on the Fast Food database in terms of (**a**) CHO, (**b**) PRO, (**c**) FAT and (**d**) Calories. The dashed lines indicate the 95% confidence interval of goFOOD^TM^ (blue) and the dietitians’ estimations (red).

To further illustrate the comparison between the performances of goFOOD^TM^ and the dietitians, the Bland–Altman plots on both databases are illustrated in Figure 7 and Figure 8. The Bland–Altman plots reveal that goFOOD^TM^ achieves a more stable performance than the dietitians on the MADiMa database, but a comparable performance on the Fast Food database.

## 5. Discussion and Conclusions

In this paper, we proposed a fully automated nutrient estimation system, goFOOD^TM^, that estimates the calorie and macronutrient content (kcal, CHO, PRO and fat) of a meal using images or video captured by smartphones. The experimental results indicate that the proposed goFOOD^TM^ system performs with a higher accuracy than the experienced dietitians on normal central-European meals, while exhibiting comparable performance for the fast food standardized meals. However, it should be noted that the study was conducted with only two dietitians from the same country. A larger number of nutrition experts with diversity backgrounds are needed in order to test the estimations again and be able to generalise our findings.

The embedded food segmentation algorithm used in this version of goFOOD^TM^ has proved to be superior to its previous version [30] and accurately recognized different foods, depending on how common and fine-grained they were. The *lowest* top-3 accuracy of 71.8% was found for the *most* fine-grained categories, which emphasizes the need for additional and more specific data, since the number of different foods that can be encountered on a daily basis is high.

The results of goFOOD^TM^ and the experienced dietitians were similar for the Fast Food database. This may be due to the dietitians’ greater familiarity with standardized meals. On the other hand, goFOOD^TM^ performed better with the MADiMa database and more real-life every day meals, which can indicate the system’s ability to generalize its findings, but which can also be dependent on the USA dietitians’ unfamiliarity with the Central European cuisine.

The above mentioned findings indicate that such an enhanced system can be of considerable practical value in different scenarios and use cases. There are a number of system versions addressing numerous use cases. goFOOD^TM^ can obviously be useful for individuals who desire to monitor their diet and be cautious either for health related reasons or for lifestyle. Similarly, it can be a very useful tool for dietitians and health care professionals that wish to monitor their patients’ or clients’ diet either by using the tool on-site or by having their patients or clients share their results. This can also further assist with producing statistical information and extracting trends, which can help in improving one’s diet. Furthermore, a simpler version of goFOOD^TM^, called goFOOD^TM^Lite, has been developed, which does not provide a meal estimation, but is simply used to record the user’s consumed foods and beverages. The data acquired using this method are appropriate for retrospective analysis. Thus, this tool is valuable to individuals in need of simple tracking of their diet and/or their dietitians or health care professionals. On a broader spectrum goFOOD^TM^Lite can be a valuable tool for large-scale data gathering research projects, both technical and nutritional, as it can be used for image-/video-data gathering. In technical research it is expensive and difficult to gather the volume of data required for the training of state of the art algorithms and for the development and research of novel methods. Similarly in nutrition research, the currently available methods for data collection are greatly cumbersome to the research participants and prone to inaccurate entries. Food recording can be tedious for participants to complete, thus constituting a reason for them not to participate in a project at all, and for researchers to analyze.

**Future Work:**

As has been made obvious throughout this work, goFOOD^TM^ is under constant development aiming at improving its current features, in terms of accuracy, speed, user friendliness and also aiming at developing and integrating new features. The most recent addition to the set of functionalities is the development and integration of a bar-code scanner, so that packaged consumed products can also be accounted for. This functionality has not been analysed and will not be further discussed in the current manuscript, as it does not constitute an active research project, but a developed package ready for integration.

Finally, only an Android app is currently available to the user. This obviously limits possible users to only those with Android phones. At the moment an iOS version is also under development so that the app can be of help to all smartphone users. The app was not available to the public when the current manuscript was drafted. Its usage is limited to research purposes, either in-lab for further development or as part of a number of research and development project that are currently ongoing.

## Figures and Tables

**Table 1:** Comparison of food segmentation results on the MADiMa database.

Methods	$F_{\min}$ (%)	$F_{\mathrm{sum}}$ (%)
[38]-Region growing & merging	67.8	90.8
[30]-$CNNborder_{segnet}$	70.6	92.9
[30]-$CNNborder_{unet}$	74.3	93.7
goFOOD^TM^	**83.9**	**94.4**

**Table 2:** Comparison of food recognition results on the MADiMa database.

Methods	Hyper1		Hyper2		Fine-Grained	
	Top-1 (%)	Top-3 (%)	Top-1 (%)	Top-3 (%)	Top-1 (%)	Top-3 (%)
Inception-V3 [32]	63.2	**83.7**	47.0	70.5	53.9	**73.6**
goFOOD^TM^	**65.8**	82.4	**61.5**	**78.2**	**57.1**	71.8

**Table 3:** Estimations of nutrient content on the MADiMa database.

	goFOOD^TM^ Median (25th– 75th Percentiles)	Dietitians Median (25th–75th Percentiles)
CHO (g)	7.2 (3.2–15.3)	27 (10.6–37.7)
PRO (g)	4.5 (2.0–10.9)	8.7 (4.7–13.5)
Fat (g)	5.2 (2.0–10.06)	5.2 (2.3–9.7)
Calories (kcal)	74.9 (40.4–139.3)	180 (119–271)

**Table 4:** Comparison of results of nutrient content estimation on the Fast Food database.

	Two-View Median (25th–75th Percentiles)	Stereo Pair Median (25th–75th Percentiles)	Dietitians Median (25th–75th Percentiles)
CHO (g)	7.9 (4.2–15.7)	9.3 (3.5–14.1)	5.3 (2.9–7)
PRO (g)	2.8 (1.3–4.20)	4.4 (2.9–7.5)	1.5 (0.5–3.3)
Fat (g)	5.8 (1.4–14.6)	9.22 (3.9–22.5)	3.8 (1.5–6.3)
Calories (kcal)	75.9 (27.9–124.7)	107.8 (54.8–150.9)	55.5 (17–83)

**Table 5:** Pearson correlations between different methods.

Database		goFOOD^TM^ vs. Ground Truth	Dietitians vs. Ground Truth	goFOOD^TM^ vs. Dietitians
MADiMa	CHO	0.54	0.57	0.40
	PRO	0.69	0.82	0.62
	Fat	0.66	0.63	0.47
	Calories	0.60	0.66	0.52
Fast Food	CHO	0.88	0.97	0.87
	PRO	0.89	0.95	0.92
	Fat	0.50	0.94	0.47
	Calories	0.87	0.97	0.83

All values of the table are p<0.05.

## Abbreviations

The following abbreviations are used in this manuscript:

AI	artificial intelligence
CHO	carbohydrate
PRO	protein

## Appendix A. High-Level Comparison between goFOODTM and Some Popular Commercial Dietary Assessment Apps

In this section, we provide a high-level comparison between the proposed goFOOD^TM^ system and some popular commercial products for dietary assessment. As mentioned in Section 1, it should be noted that the commercial products normally do not provide the details regarding the algorithms used and validation results. Thus, a systematic and in depth comparison is not feasible (e.g., there is no way for us to know the configuration of their products in the sense of algorithmic execution on the server side or smartphone side). Nevertheless, Table A1 summarises the major differences and advantages of the proposed approach (goFOOD^TM^) against commercial products.

**Table A1:** High-level comparison between goFOOD^TM^ and some popular commercial dietary assessment apps.

APP name ^1^	Automatic Food Recognition	Automatic Food Portion Estimation	Core Algorithm	Computa- Tional Time ^2^	Validation
FatSecret [27]	×	×	-	<1 s	-
CALORIE MAMA [28]	*√*	×	CNN for food recognition	<1 s	-
bitesnap [29]	*√*	×	CNN for food recognition	<1 s	-
aical [42]	*√*	×	Voice recognition aided food recognition	<1 s	-
GoCARB ^3^ previous version of goFOOD^TM^	*√*	*√*	Traditional machine learning for food recognition, segmentation; SfM for food 3D model reconstruction (volume calculation)	∼2 s for food recognition and segmentation; ∼5 s for volume estimation	· Technical · Pre-clinical · Clinical
goFOOD^TM 4^	*√*	*√*	CNN for food recognition, segmentation; Improved SfM for food 3D model reconstruction (volume calculation)	<1 s for food recognition and segmentation; ∼2 s for food volume estimation	· Technical · Pre-clinical ongoing, 2020

1. These commercial apps are chosen because of their high number of downloads; 2. It is impossible for us to know the precise running time of the commercial apps, thus we chose 1s as the threshold; 3. https://gocarb.ch; 4. https://go-food.tech.

## Appendix B. Macronutrient Estimation from the Dietitians

The goal was to calculate the nutritional content in terms of energy and macronutrients (CHO, PRO and fat) for each of the food items in every plate. The nutritional content of each of the 80 dishes was calculated using the USDA nutritional database, the Swiss nutrient database [37] or the food label referring to each food item. Certain images contained meals consisting of several food items, but whose food labels did not provide nutritional information for each separate item, but for the entire meal. For these meals, the USDA nutritional database or the Swiss nutrient database were used, where needed, in order to calculate each food item’s nutritional content, with a precision as close to the labeled food as possible.

When specific food items did not exist as separate food items, either on the food labels or in the nutrient databases (e.g., curry chicken with pasta written on the label, but no individual nutritional values for chicken with curry sauce and pasta), then a professional dietitian estimated the amount of sauce and the amount of meat that was present on the plate and calculated the nutritional content of the sum of those food items accordingly.

## Appendix C. Food Categories

The food categories supported by goFOOD^TM^ are structured in a three-level hierarchical scheme. These three levels are divided into two levels of hyper-categories and one level of fine grained categories. A visual presentation of the three-level is provided in Figure 4. These three levels contain finer food categories while moving “downwards”. Starting from the first level, we mainly have basic food groups. Moving to the second level we have a sub-categorization of these basic food groups (e.g., past can be divided into white, non white and stuffed past). Finally, the fine categories of the third level contain actual dishes with main ingredient the one from the corresponding second or first level. In Table A2 we provide the full list of the first two levels, while also some indicative examples of the finer third level categories. Certain categories exist in both the first and second level (e.g., “rice” as a 1st level hyper-category and as a 2nd level category, under “Mixed”). This separation is based on the fact that there are certain dishes that contain a food type in high quantities, but also contain other types in such ratios, so that the first type cannot constitute the category, but a main ingredient of a mixed dish. For example, the dish “bibimbap” contains rice in a large quantity, enough to consider it the main/basic ingredient. But it also contains other ingredients (vegetables, meat, egg) and in such quantities that the entire dish is considered as mixed.

Our system is trained on all levels and can recognize the food as well as its two broader categories. In the case that an image contains a dish, which is not included in the 319 fine categories, the system can provide the second and first level categories, along with their nutrient content. This way, the user can receive the most accurate estimation possible.

**Table A2:** goFOOD^TM^ Food Cateogories.

1st Level	2nd Level	Fine Categories
Bread	white	garlic bread
	non white	
Pasta	white	couscous, spaghetti, penne with tomato sauce, etc.
	non white
	stuffed pasta	ravioli, spinach tortellini, etc.
Potatoes	None	french fries, boiled potatoes with skin, etc.
Pulses/legumes	None	peas, poi, etc.
Rice	white	pilaf, etc.
	non white	wild rice, etc.
Fish and Seafood	None	oyster, clam food, lutefisk, etc.
Fruit	None	acerolas, pineapples, apples, etc.
Meat	processed products	sausage products, galantine, etc.
	white meat	fried chicken, creamy chicken, turkey with cheese, etc.
	red meat	meatballs, steak au poivre, etc.
Dairy products	yoghurt	plain yoghurt, mixed yoghurt
	white cheese	hard white cheese, cottage cheese, etc.
	yellow cheese	fondue
Eggs	boiled/baked	boiled egg, deviled egg, etc.
	fried	omelette, frittata, etc.
Sweets	None	churro, panna cotta, flan, etc.
Vegetables	None	carrots, mushrooms, string beans, etc.
Mixed	gratins	casserole, ziti, tamale pie, etc.
	salads	green salad, beet salad, seaweed salad, etc.
	open sandwiches	tostada, bruschetta, huevos rancheros, etc.
	closed sandwiches	hamburger, lobster roll sandwich, club sandwich, etc.
	stuffed food	dumpling, burrito, gyoza, etc.
	pizza	
	multilayer	lasagna, moussaka, etc.
	soup	wonton, pho, miso soup, etc.
	noodles/pasta	chow mein
	rice	biryani, pad thai, bibimbap, etc.
	meat	coq au vin, moo moo gai pan, etc.
	fish	lobster thermidor, fish and chips, etc.
	other	kedgeree, guacamole, sushi, etc.
Breaded (incl. croquettes)	None	falafel, tempura, samosa, etc.
Corn	None	
Nuts	None	pecan, hazelnut, etc.
Snack	None	chips, nachos, etc.
Cereal	processed	
	unprocessed	
